# Riding a Vascular Time Train to Spatiotemporally Attenuate Thrombosis and Restenosis by Double Presentation of Therapeutic Gas and Biomacromolecules

**DOI:** 10.1002/EXP.70004

**Published:** 2025-02-04

**Authors:** Jingdong Rao, Di Suo, Qing Ma, Yongyi Mo, Ho‐Pan Bei, Li Wang, Chuyang Y. Tang, Kai‐Hang Yiu, Shuqi Wang, Zhilu Yang, Xin Zhao

**Affiliations:** ^1^ Department of Applied Biology and Chemical Technology The Hong Kong Polytechnic University Hong Kong SAR China; ^2^ Department of Biomedical Engineering The Hong Kong Polytechnic University Hong Kong SAR China; ^3^ The Hong Kong Polytechnic University Shenzhen Research Institute Shenzhen China; ^4^ Dongguan Key Laboratory of Smart Biomaterials and Regenerative Medicine The Tenth Affiliated Hospital of Southern Medical University Dongguan Guangdong China; ^5^ Guangdong Provincial Key Laboratory of Cardiac Function and Microcirculation Guangzhou Guangdong China; ^6^ Department of Civil Engineering The University of Hong Kong Hong Kong SAR China; ^7^ Cardiology Division, Department of Medicine The University of Hong Kong, Queen Mary Hospital Hong Kong SAR China; ^8^ Tianfu Jincheng Laboratory City of Future Medicine Chengdu China; ^9^ College of Biomedical Engineering, Sichuan University Chengdu China; ^10^ Research Institute for Intelligent Wearable Systems The Hong Kong Polytechnic University Hong Kong SAR China; ^11^ Research Institute for Future Food The Hong Kong Polytechnic University Hong Kong SAR China

**Keywords:** anti‐restenosis, anti‐thrombosis, biomacromolecule, re‐endothelialization, therapeutic gas

## Abstract

Endothelial injury is a common occurrence following stent implantation, often leading to complications such as restenosis and thrombosis. To address this issue, we have developed a multi‐functional stent coating that combines a dopamine‐copper (DA‐Cu) base with therapeutic biomolecule modification, including nitric oxide (NO) precursor L‐arginine, endothelial glycocalyx heparin, and endothelial cell (EC) catcher vascular endothelial growth factor (VEGF). In our stent coating, the incorporated Cu acts as a sustainable catalyst for converting endogenous NO donors into NO, and the immobilized arginine serves as a precursor for NO generation under the effect of endothelial nitric oxide synthase (eNOS). The presence of heparin endows the stent coating with anticoagulant ability and enhances eNOS activity, whilst rapid capture of EC by VEGF accelerates re‐endothelialization. After in vivo implantation, the antioxidant elements and produced NO alleviate the inflammatory response, establishing a favorable healing environment. The conjugated VEGF contributes to the formation of a new and intact endothelium on the stent surface to counteract inappropriate vascular cell behaviors. The long‐lasting NO flux inhibits smooth muscle cell (SMC) migration and prevents its excessive proliferation, reducing the risk of endothelial hyperplasia. This innovative coating enables the dual delivery of VEGF and NO to target procedural vascular repair phases: promoting rapid re‐endothelialization, effectively preventing thrombosis, and suppressing inflammation and restenosis. Ultimately, this innovative coating has the potential to improve therapeutic outcomes following stent implantation.

## Graphical abstract

This project develops a biomimetic stent coating with a double presentation of therapeutic biomacromolecule VEGF and therapeutic gas NO, which spatiotemporally promotes vessel repair including promoting rapid re‐endothelialization, effectively preventing thrombosis, suppressing inflammation and restenosis.

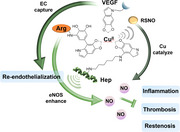

## 1 Introduction

Cardiovascular stent (CVS) implantation is the mainstay treatment for cardiovascular diseases (CVDs) [1]. However, the stent implantation process unavoidably causes mechanical damage to the vascular endothelium, leading to disturbance of the vascular microenvironment, prolonged inflammatory response and delayed re‐endothelialization. As a natural barrier, the endothelium can impede unfavorable cellular behaviors such as pro‐inflammatory cell dysregulation, activated platelet adherence and excessive smooth muscle cell (SMC) proliferation. When the endothelium loses its integrity, the above‐mentioned behaviors occur, leading to thrombosis and restenosis [2]. Consequently, to restore vascular homeostasis effectively, an ideal CVS should prioritize endothelial repair and address all facets of cellular events following stent implantation.

In natural vascular endothelium, the presence of nitric oxide (NO) and glycosaminoglycan maintains an anti‐inflammatory and anticoagulant environment. NO, a versatile gas molecule secreted by endothelial cells (ECs) [3], plays a crucial role in promoting a harmonious vascular environment [4]. It can enhance EC growth, reduce macrophage pro‐inflammatory polarization [5], and inhibit platelet adhesion and SMC proliferation [6]. However, in an inflammatory environment, NO can be consumed and deactivated by reactive oxygen species (ROS), leading to reduced bioavailability and NO levels [7]. Inflammation can also hinder NO production by uncoupling endothelial NO synthase (eNOS). Additionally, the polarized M1 phenotype macrophages will secrete proinflammatory factors, leading to endothelial dysfunction and delayed re‐endothelialization [8]. Long‐term inflammatory response further promotes glycoprotein deposition, SMC hyperproliferation, and thrombosis [2, 9]. The glycocalyx (particularly heparin), a prominent component on the endothelial surface, not only inhibits reactive ROS production but also promotes NO release from NO precursor L‐arginine by enhancing the activity of eNOS [10]. The incorporation of heparin and NO precursors into stents can alleviate inflammation and thrombosis [11], however, the rapid recruitment of ECs to promote early re‐endothelialization remains challenging, and incomplete endothelium provides a chance for the invasion of platelets and SMCs [12]. VEGF has been identified as a key mediator in cell‐matrix interactions [13], where ECs can directly adhere to and spread during angiogenesis [14]. Additionally, the binding of VEGF to EC receptors (including α3β1 and αvβ3 integrins) activates a number of important intracellular signaling molecules to mediate EC migration, proliferation and tubule formation [15]. Hence, the combination of VEGF with glycocalyx‐associated NO‐producing stent coating holds great potential in alleviating inflammatory response and promoting EC adherence to facilitate re‐endothelialization, suppress platelet adhesion and SMC proliferation, and ultimately inhibit thrombosis and restenosis.

**FIGURE 1:**
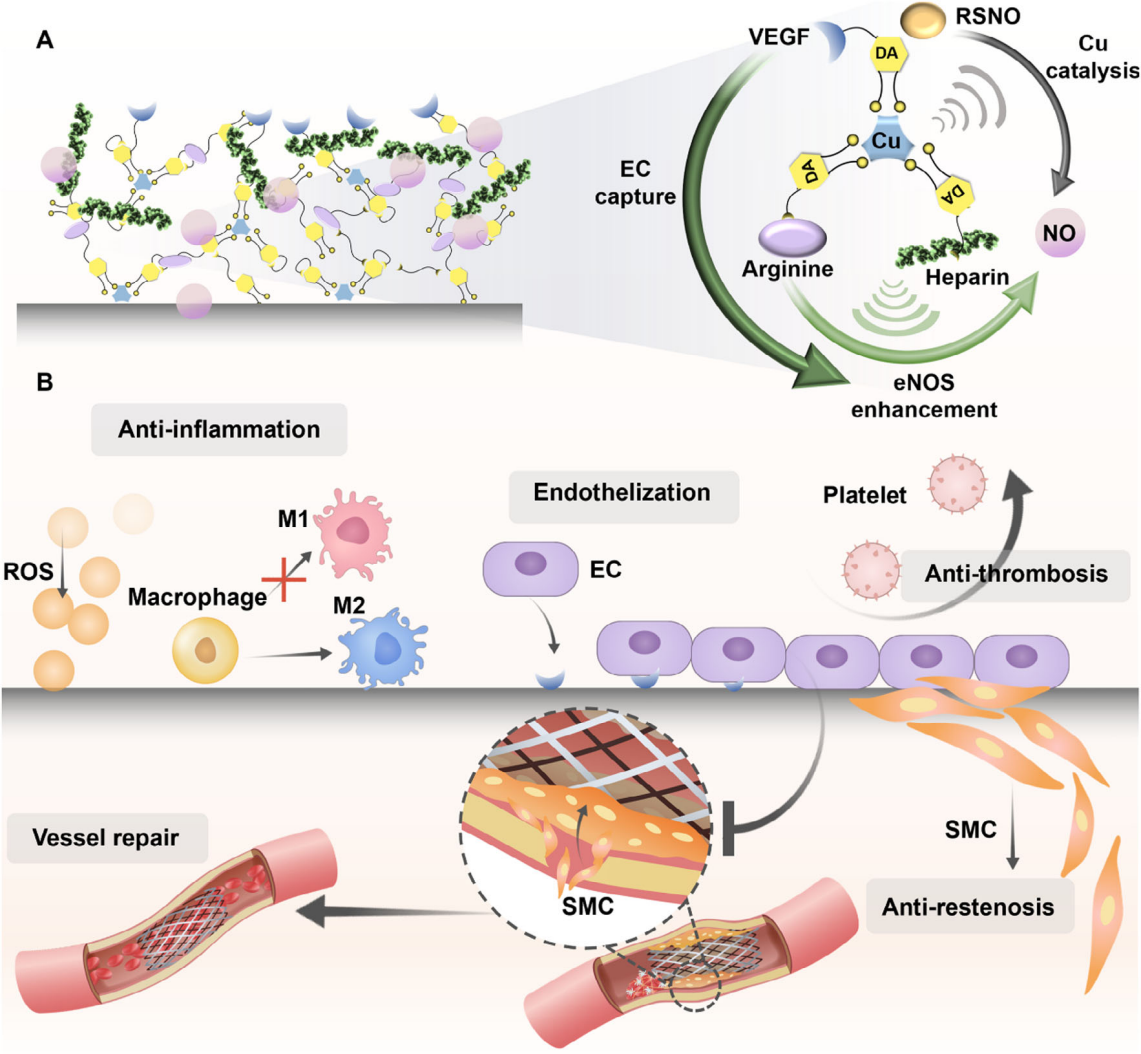
Illustration of spatiotemporal therapeutic effect of the AHV coating. (A) The AHV coating is prepared by an immersion approach, where the DA‐Cu network adheres to the stent surface with functional molecular modifications. The grafting of VEGF facilitates the efficient capture of ECs. Simultaneously, Cu catalyzes the NO production when endogenous RSNOs were present, while in the scenarios where RSNOs are absent, the NO precursor arginine plays a vital role in NO synthesis. The conversion of arginine into NO is mediated by eNOS, whose level is influenced by the increasing number of ECs and can be further elevated by heparin. Such interplay ensures the production of sufficient NO under various physiological conditions. (B) After in vivo implantation of the AHV stent, the antioxidants DA, heparin and L‐arginine reduce ROS level, and then polarize macrophages into anti‐inflammatory M2 type with the assistance of generated NO, heparin and VEGF, alleviating local inflammation. Afterward, the grafted VEGF promotes rapid re‐endothelialization, enabling the restoration of endothelial functions and establishing a barrier against inappropriate cellular behaviors. The produced NO and anticoagulant heparin can synergistically inhibit platelet adhesion and activation, preventing thrombosis. The sustained NO production also suppresses SMC migration and proliferation at the later stage of stent implantation for a long time, reducing the risk of restenosis and promoting vessel repair. Altogether, the AHV coating provides a sequential treatment for the post‐implantation complications associated with stenting. EC: endothelial cell, VEGF: vascular endothelial growth factor, RSNO: S‐nitrosothiols, Cu: copper, DA: dopamine, eNOS: endothelial nitric oxide synthase, NO: nitric oxide, ROS: reactive oxygen species, M1: polarized M1 phenotype macrophages (pro‐inflammatory), M2: polarized M1 phenotype macrophages (anti‐inflammatory), SMC: smooth muscle cell.

Previous studies have developed various bio‐functional molecule delivery stents, such as NO‐catalyzing stents [16], NO prodrug stents [17], drug‐eluting stents [18], VEGF gene stents [19], nanoparticle stents [20] and so on, all of which have shown promising therapeutic effects, including re‐endothelialization and anti‐hyperplasia. However, limitations such as shortage of NO donors, short‐term availability of VEGF and limited effect of monotherapy pose challenges to clinical translation [10, 11]. Therefore, there is an urgent need for the development of stents that can address the deficiency of NO supply and deliver multiple bio‐functional molecules. Here, we lay the foundation for re‐endothelialization on CVS by fabricating a stent coating called AHV coating, which consists of a mussel‐inspired dopamine (DA)/hexamethylenediamine (HD)‐copper (Cu) network grafted with the NO precursor L‐arginine, endothelial glycocalyx heparin and EC catcher VEGF. After in vivo implantation, the AHV coating quickly eliminates ROS in the inflammatory microenvironment by the antioxidant properties of DA, heparin and arginine, ensuring the physiological effects of generated NO. It has been reported that Cu has GPx‐like activity in terms of NO production, as Cu(II) can be reduced into Cu(I) to decompose endogenous NO donors S‐nitrosothiols (RSNOs) into NO [21]. In the absence of RSNOs, the NO precursor arginine plays a crucial role in NO production under the eNOS‐enhancing effect of heparin. This synergistic effect ensures a sustained and sufficient supply of NO, compensating for the potential reduction of RSNO levels in the actual pathophysiological environment. Afterward, the produced NO directs macrophages into the anti‐inflammatory M2 type, and the elimination of ROS and favorable macrophage polarization provide a healthy environment for blood vessel repair. Meanwhile, the presence of VEGF facilitates rapid EC adhesion, accelerating re‐endothelialization and forming a protective barrier on stent surface against detrimental cellular behaviors. Then, the heparin and NO reduce platelet adhesion and maintain an anticoagulant environment, reducing thrombosis risk. Furthermore, the continuous production of NO can diffuse out and permeate into SMC membranes to activate soluble guanylate cyclase (sGC) and enhance cyclic guanylate monophosphate (cGMP) expression, suppressing intimal hyperplasia and expanding blood vessel lumen in the long‐term, ultimately promoting repair of an injured blood vessel (Figure 1).

Altogether, the composition of the AHV coating adapts to the progression of blood vessel repair, each corresponding to cellular events after stenting. Such sequential responses ensure that the stent addresses the evolving needs of the surrounding tissues. Furthermore, our stent coating has a spatial impact on the blood vessel environment. At the vessel wall, the over‐growth and migration into the lumen of SMCs are inhibited, effectively reducing vessel narrowing. Within the vessel lumen, the rapid re‐endothelialization and reduction of thrombus deposition restore proper blood flow, reshaping a healthy endothelial environment. This innovative coating holds promise for improving clinical outcomes and reducing complications associated with CVS implantation.

## 2 Results and Discussion

### 2.1 Characterization of AHV Coatings

**FIGURE 2:**
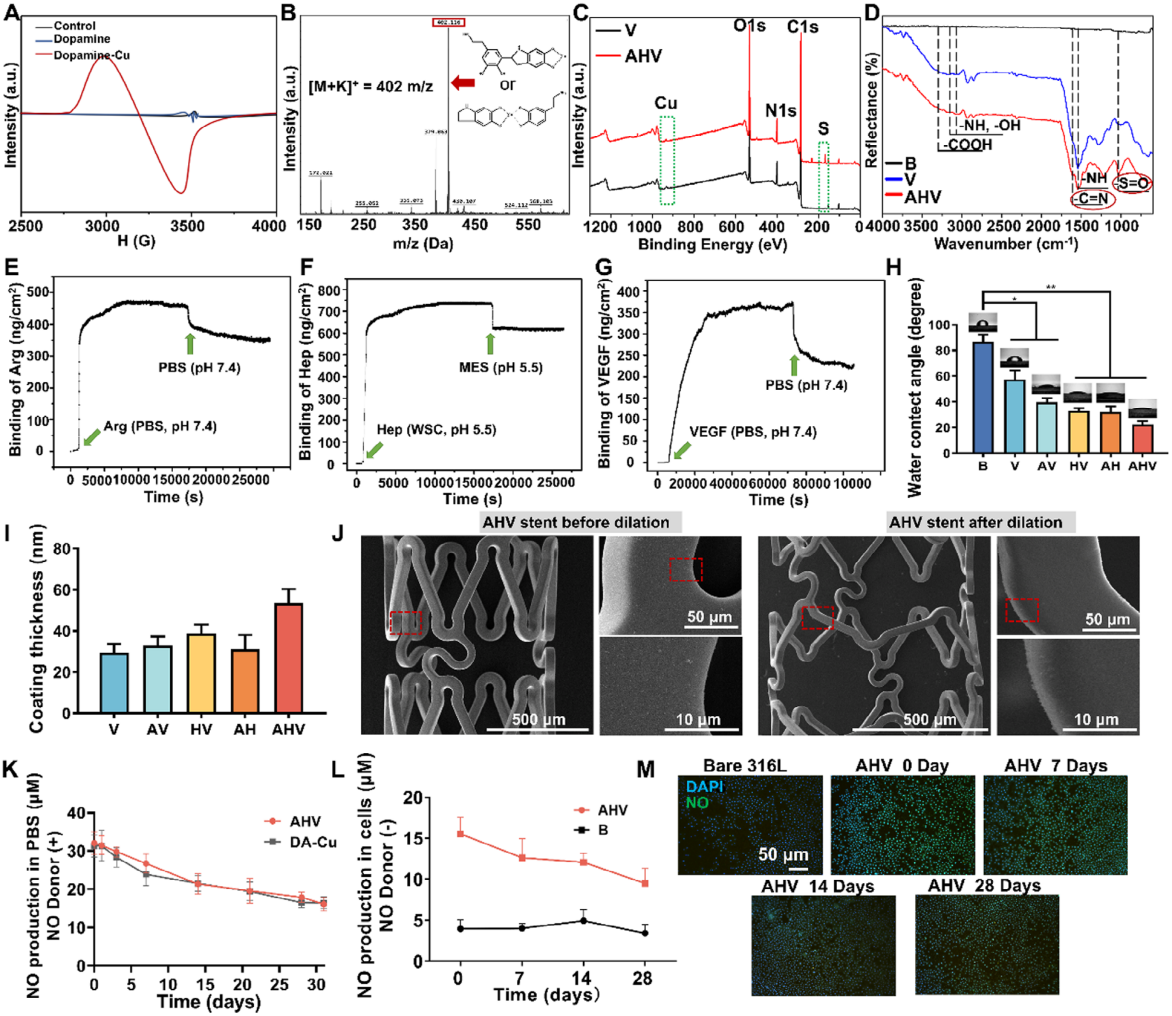
Characterization of the AHV coating. (A) EPR and (B) MALDI‐TOF‐MS spectra of DA‐Cu. (C) XPS spectra of V and AHV. (D) GATR‐FTIR spectra of B, V and AHV. Immobilization of (E) arginine, (F) heparin and (G) VEGF onto the DA/HD‐Cu coating measured using QCM‐D. Approximately 346 ± 11 ng cm^−2^ arginine, 609 ± 7 ng cm^−2^ heparin and 232 ± 4 ng cm^−2^ VEGF had been conjugated onto the sample surface. (H) WCA of bare and coated samples. (I) Thickness of each coating. (J) Representative SEM micrographs showing the morphology of the AHV‐coating before and after stent dilation. Scale bars: 10, 50 and 500 µm. (K) Long‐term NO generation with NO donors. (L) NO production by arginine in cellular level without NO donors. (M) NO production (green) by arginine in HUVECs (with no NO donors). Scale bars: 50 µm. Data were displayed as mean ± standard deviation (SD) (*n* = 3). *p < 0.05 and **p < 0.01. B: bare 316L SS, DA: dopamine, Cu: copper, V: DA/hexamethylenediamine (HD)‐Cu‐VEGF, AV: DA/HD‐Cu‐arginine‐VEGF, HV: DA/HD‐Cu‐heparin‐VEGF, AH: DA/HD‐Cu‐arginine‐heparin, AHV: DA/HD‐Cu‐arginine‐heparin‐VEGF, PBS: phosphate buffered saline, WSC: water soluble carbodiimide, MES: 2‐(*N*‐morpholino) ethanesulfonic acid hydrate, NO: nitric oxide, DAPI: 4',6‐diamidino‐2‐phenylindole.

The AHV coatings were prepared on the 316L stainless steel (316L SS, one of the most common materials used for CVS) substrates by an organic‐free dipping method [10c]. Bare 316L SS (B), DA/HD‐Cu‐VEGF (V), DA/HD‐Cu‐arginine‐VEGF (AV), DA/HD‐Cu‐heparin‐VEGF (HV), and DA/HD‐Cu‐arginine‐heparin (AH) served as control groups. Electron paramagnetic resonance (EPR) spectroscopy was employed to investigate the coordinated reaction between DA and Cu. Figure 2A reveals a negligible signal of DA, while a significant Cu‐related signal at around 3400 mT was observed in DA‐Cu, indicating successful coordination of Cu and DA. Then, to investigate the polymerization mechanism, a matrix‐assisted laser desorption ionization mass spectrometry (MALDI‐TOF‐MS) study was carried out (Figure 2B), and the peaks observed at 402 m z^−1^ suggested potential structures of DA‐Cu, further confirming the chelation of Cu and DA derivatives. Subsequently, X‐ray photoelectron spectroscopy (XPS) was adopted to analyze the chemical elements (Figure 2C). The data demonstrated a strong Cu signal in all groups, while the AHV group exhibited an additional S signal (from heparin). To investigate the chemical structure of AHV coating, attenuated total reflection Fourier transform infrared spectroscopy (ATR‐FTIR) was also used. In Figure 2D, the fundamental structure of each coating was identical (with the same ─COOH, ─NH, ─OH and ─C═C peaks), demonstrating the formation of the DA network. In AHV samples, ─C═N bond and ─S═O bond were detected by the peaks around 1600 and 1000 cm^−1^, indicating the successful modification with arginine and heparin. Subsequently, the grafting amount of arginine, heparin, and VEGF was quantified by quartz crystal microbalance with dissipation (QCM‐D). Figure 2E–G illustrates that ≈346 ± 11 ng cm^−2^ arginine, 609 ± 7 ng cm^−2^ heparin and 232 ± 4 ng cm^−2^ VEGF had been conjugated onto the sample surface, confirming the successful fabrication of the AHV coating.

Afterward, the physical properties of the AHV coating were studied. The water contact angles (WCA) of each sample were first tested to evaluate the wettability of the coatings [21]. The bare samples exhibited a high WCA of approximately 85°, suggesting its surface was hydrophobic. The introduction of arginine, heparin, and VEGF resulted in a significant decrease in WCA (around 23° in AHV), indicating improved hydrophilicity that potentially benefits cell adhesion (Figure 2H). In addition, the coating thickness of all samples was between 30 and 60 nm (Figure 2I), which is expected to be stable due to the nanometer thickness [22]. To assess the mechanical stability of AHV coating, a dilated balloon angioplasty procedure was performed [16b]. The scanning electron microscopy (SEM) results showed the AHV coating had no delamination and cracks after dilation, indicating appropriate mechanical properties of the coating (Figure 2J). Additionally, we observed the dilated AHV stents subjected to 30 days of flush with phosphate buffer saline (PBS) by SEM (Figure S1). We found that the coated stent displayed a uniform coating with no visible cracks. Energy dispersive X‐ray (EDX) spectroscopy demonstrated obvious N, O, C, Cu and S distribution in the AHV coating even after the long‐term flush, confirming its robust stability. The above results demonstrated that the coating exhibited superior mechanical strength and chemical stability: it remained intact without peeling and maintained its chemical structure after a long‐term PBS wash.

### 2.2 In Vitro Long‐Term NO Production

The AHV coating had two pathways to produce NO. The first pathway involved Cu catalysis, where the reduction of Cu(II) to Cu(I) facilitated the production of NO from NO donors such as RSNOs or S‐nitroso‐N‐acetylpenicillamine (SNAP) under the effect of glutathione (GSH), while forming byproduct glutathione disulfide (GSSG) [23]. The second pathway relied on the decomposition of arginine. In the presence of oxygen and NADPH, arginine could be converted into NO through the action of eNOS in ECs, whose activity can be increased by heparin, and its byproducts (citrulline) would undergo recycling into arginine [24]. The synergistic combination of the catalytic Cu, the NO precursor arginine, and the eNOS enhancer heparin enables sustained NO production under any physiological condition regardless of the presence of NO donors, thereby avoiding NO deficiency due to the depleted RSNOs. To investigate the AHV coating's potential for producing NO by Cu catalytic effect, the DA/HD‐Cu and AHV samples were immersed in pH 7.4 PBS solution containing NO donors. Figure 2K shows that both groups produced large amounts of NO, with no obvious distinction between DA/HD‐Cu and AHV, suggesting surface grafting had no influence on NO production. The AHV samples maintained the long‐term NO production, generating about 50.15% of their initial NO production at 30 days. Furthermore, to investigate the NO production from the AHV coating through the NO precursor arginine, samples soaked in PBS were taken out at day 0, 7, 14 and 28, and human umbilical vein endothelial cells (HUVECs) were seeded onto its surfaces without NO donors. The quantitation data (Figure 2L) showed that the AHV coating could support long‐term NO generation at the cellular level in the absence of NO donors, which was attributed to the existence of arginine. Fluorescence images (Figure 2M) revealed a lack of NO signal in the bare samples, while the AHV samples exhibited strong green fluorescence for 28 days, confirming the continuous NO production from AHV. The detected total NO flux rate of the AHV coating with and without NO donors were 3.56 and 1.81 × 10^−10^ mol cm^−2^ min^−1^ respectively, which were comparable to the physiological NO rate (0.5–4 × 10^−10^ mol cm^−2^ min^−1^) [25]. Overall, these results validated that the AHV coating could generate NO persistently, independent of NO donors, which suggested its potential therapeutic effectiveness in the long‐term prevention of stent restenosis and thrombosis.

### 2.3 In Vitro Biological Properties of AHV Coatings

#### 2.3.1 Inflammation Alleviation

**FIGURE 3:**
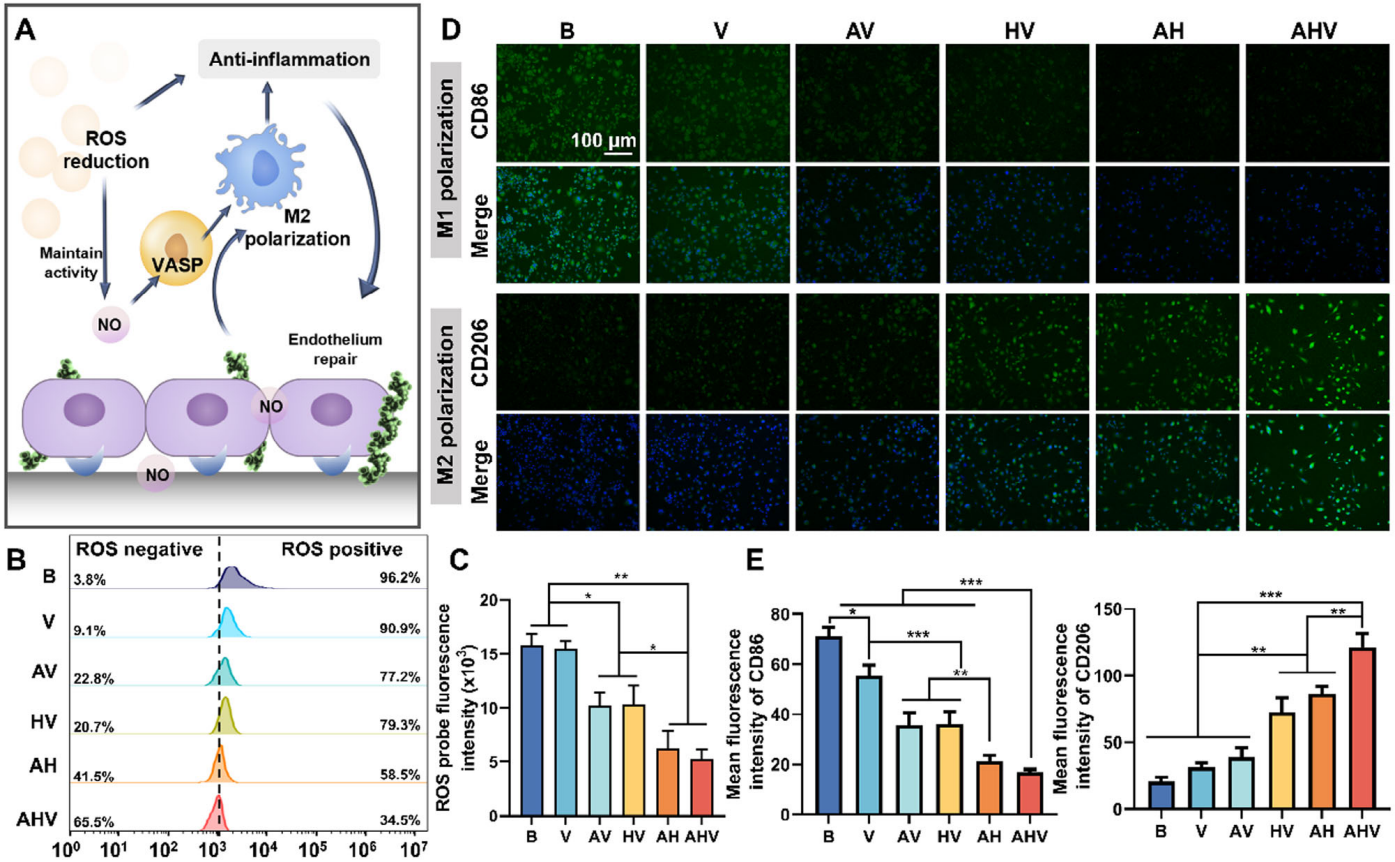
Inflammation alleviation. (A) Illustration of rapid inflammation alleviation. (B) Histogram and (C) fluorescence quantification of ROS scavenging. (D) Immunofluorescence staining of macrophage polarization (top: CD86, M1 marker; bottom: CD206, M2 marker) and (E) semi‐quantitative data. Scale bars: 100 µm. Data were displayed as mean ± standard deviation (SD) (*n* = 3). *p < 0.05, **p < 0.01, ***p< 0.001. NO: nitric oxide, ROS: reactive oxygen species, VASP: vasodilator stimulated phosphoprotein, M1: polarized M1 phenotype macrophages (pro‐inflammatory), M2: polarized M1 phenotype macrophages (anti‐inflammatory), B: bare 316L SS, V: DA/HD‐Cu‐VEGF, AV: DA/HD‐Cu‐arginine‐VEGF, HV: DA/HD‐Cu‐heparin‐VEGF, AH: DA/HD‐Cu‐arginine‐heparin, AHV: DA/HD‐Cu‐arginine‐heparin‐VEGF.

Stent implantation causes mechanical damage to the endothelium, triggering local inflammation. Following inflammation, macrophages accumulate, adhere to the stent surface and release pro‐inflammatory cytokines [8b]. Meanwhile, the production of ROS, a key marker of inflammation, not only leads to endothelial dysfunction but also inactivates NO production and reduces its bioavailability [26]. These inflammatory responses further contribute to glycoprotein deposition, SMC hyperproliferation and thrombosis [27]. Therefore, it is crucial to mitigate the inflammatory environment following stent placement. In our proposed AHV coating, the antioxidants DA, heparin and arginine can effectively reduce ROS levels and preserve the physiological activity of NO. This, in turn, leads to the polarization of macrophages into the anti‐inflammatory M2 type through the immunomodulation of NO, heparin and VEGF, synergistically alleviating the local inflammation (Figure 3A).

We first evaluated the ROS level of each sample. In the lipopolysaccharide (LPS)‐induced inflammation model, the bare group showed a strong ROS signal, manifesting high fluorescence intensity and a rightward shift of the histogram, while the coating groups demonstrated varying degrees of ROS reduction due to the presence of ROS scavengers (Figure 3B,C). Among all groups, the AHV exhibited significant reductions in ROS levels, which shifted 65.5% peak to the ROS elimination area, and reduced ROS levels by 3.02‐fold compared with the B group. The decreased ROS level contributed to the alleviation of the inflammatory environment and maintained the physiological effects of NO. It has been reported that NO is capable of polarizing macrophages toward an anti‐inflammatory M2 phenotype [5]. The immunofluorescence and semi‐quantitative results (Figure 3D,E) demonstrated the macrophages turned to M1 polarization on the bare surface as they displayed significant M1 marker and weak M2 marker. Coated samples showed a tendency toward M2 polarization, where AHV had the strongest M2 signal. Meanwhile, a significant down‐regulation of the pro‐inflammatory marker TNF‐α was found in the HV, AH and AHV groups, while the anti‐inflammatory marker IL‐10 was remarkably enhanced in the AH and AHV groups (Figure S2A,B).

It is worth noting that the morphology of macrophages underwent significant changes because the polarization leads to alterations in cell shape [28], with the bare group showing mostly flat pancake‐like cells (M1) while the coated group displayed varying degrees of elongation and spindle morphology (M2) (Figure S2C). The highest number of spindle‐shaped cells could be found in the AHV group, further highlighting its ability to promote anti‐inflammatory polarization. The strongest anti‐inflammatory outcomes observed in the AHV group were attributed to its pleiotropic abilities including the potent production of NO, the immunomodulatory influences of heparin and the potential M2‐directed impacts of VEGF, synergistically promoting the anti‐inflammatory effects [29]. These regulations reduced the potential threats to vascular repair and created a healthy microenvironment for the following endothelialization, anti‐thrombosis and anti‐restenosis properties.

#### 2.3.2 Enhanced Re‐Endothelialization and Platelet Repelling

**FIGURE 4:**
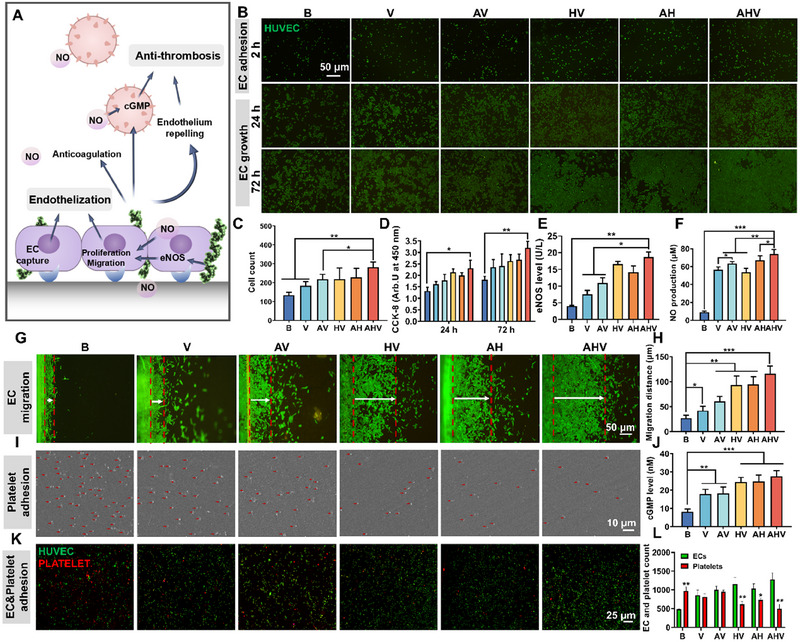
Enhanced re‐endothelialization and platelet repelling effect. (A) Illustration of EC growth and platelet inhibition. (B) The rapid adhesion and growth of HUVECs (green). Scale bars: 50 µm. (C) Number of adhered HUVECs on different coatings. (D) HUVEC proliferation using CCK‐8 assay. (E) eNOS expression and (F) NO production in HUVECs after incubation for 72 h. The increased EC adhesion, enhanced eNOS level and NO production contribute to the enhanced EC growth and rapid re‐endothelialization. (G) Fluorescence images of migrated HUVECs and (H) migration distance of HUVECs. Scale bars: 50 µm. (I) SEM images showing the adhered platelets (red arrows represent platelets) and (J) cGMP level of platelets with NO donors. Scale bars: 10 µm. (K) HUVEC and platelet (bottom) adhesion onto different coatings. AHV surface has the highest HUVEC adhesion with the lowest platelet adhesion. Scale bars: 25 µm. (L) The number of HUVECs and platelets cultivated on different coatings. Data were displayed as mean ± standard deviation (SD) (*n* = 3). *p < 0.05, **p < 0.01, ***p< 0.001. NO: nitric oxide, cGMP: cyclic guanylate monophosphate, eNOS: endothelial nitric oxide synthase, HUVEC: human umbilical vein endothelial cell, B: bare 316L SS, V: DA/HD‐Cu‐VEGF, AV: DA/HD‐Cu‐arginine‐VEGF, HV: DA/HD‐Cu‐heparin‐VEGF, AH: DA/HD‐Cu‐arginine‐heparin, AHV: DA/HD‐Cu‐arginine‐heparin‐VEGF, CCK‐8: cell counting kit‐8.

Vascular endothelium is critical in maintaining an antithrombotic and anticoagulant environment. After stent implantation, rapid adhesion of ECs provides necessary conditions for EC proliferation and migration to achieve early‐stage re‐endothelialization, and the formation of an endothelium will serve as a protective barrier against platelet adhesion (Figure 4A) [30]. To assess the impact of the AHV coating on rapid EC adhesion, HUVECs were seeded on the developed coatings for 2 h. Consistent with previous studies, we observed that the surface modified with VEGF promoted EC adhesion compared to the bare surface. Interestingly, both AH and HV coatings exhibited similar EC adhesive behavior, which could be attributed to the positive charge of arginine (Figure 4B,C). Among all groups, the AHV group showed the highest cell number (280 ± 21 cells), which was 2.02‐ and 1.62‐fold higher than the B and AH groups, respectively, indicating AHV had the potential to promote early‐stage re‐endothelialization owing to the modification of VEGF. Afterward, the proliferation and migration of HUVECs on the developed coatings were evaluated. In Figure 4B,D, the bare surface had limited EC growth, while the AHV coating was fully covered by HUVECs with a 1.77‐fold higher proliferation rate than the bare surface. These findings proved that the synergistic effect of each element in the AHV coating could promote EC growth. Additionally, it was found that with the introduction of arginine, the influence of the NO donors was weakened as indicated by the decreased differences between each group, which suggested the dependence of stent coating on NO donors was reduced.

The mechanism of increased EC growth was further investigated by evaluating the expression of eNOS, an important functional protein in ECs involved in its proliferation and arginine decomposition [31]. In Figure 4E, AV had a higher eNOS level than V, possibly because arginine possesses the potential to enhance eNOS expression [32]. It has been reported that heparin can elevate eNOS expression [2a], and our results proved that all the heparin‐containing groups showed higher eNOS levels, which was beneficial for NO production. Moreover, the rapid adhesion of EC by VEGF provides the necessary arena to fully exert the coating effects, shown as the HV group (16.55 ± 0.52 U L^−1^) had higher eNOS level than the AH group (14.13 ± 1.22 U L^−1^). Among all the groups, the AHV had the highest eNOS expression (18.71 ± 1.10 U L^−1^) due to the synergistic effects of VEGF‐mediated EC capture, arginine and heparin's eNOS‐enhancing ability.

The enhanced eNOS level contributes to arginine decomposition into NO. Subsequently, NO quantification was carried out. As shown in Figure 4F, the amount of generated NO was boosted in all coated groups, and the arginine‐containing groups showed higher NO production (AV, AH and AHV) compared with the group without arginine (V and HV). It is worth noting that although HV exhibited a higher eNOS level than AH, the NO production of HV dropped to 50.68 ± 4.11 µM (AH: 64.14± 4.50 µM NO) due to the absence of NO precursor arginine. The highest NO amount was found in the AHV group, reaching 74.17 ± 4.82 µM, which was eight‐fold higher than that of the bare group. To better understand the NO production ability of arginine, an eNOS inhibition experiment was conducted using the NOS inhibitor L‐NMMA (Figure S3). The results revealed that after adding the L‐NMMA, the eNOS levels decreased significantly and subsequently impaired the NO production in all groups. However, in the absence of eNOS inhibitors, the expression of eNOS was restored with the most pronounced increase observed in the heparin‐containing group. This restoration of the eNOS expression corresponded to a significant increase in the NO production, particularly in the AH and AHV groups. These results confirmed that arginine contributed to the NO production, and with the synergistic effects of VEGF‐mediated cell capture and heparin‐induced eNOS upregulation, the AHV group exhibited the highest level of NO production. Such increased eNOS expression and potent NO production could facilitate EC growth.

For in situ re‐endothelialization following stenting, it is essential to encourage EC migration from nearby endothelial tissues to the stent surface. Here, the cell migration on different coatings was evaluated. In Figure 4G,H, the bare group showed limited migration of ECs (around 26.7 µm). In contrast, the AHV group showed observably enhanced cell migration, with a distance of 116.4 µm, nearly five times that of the bare samples. Consequently, owing to the increased EC adhesion, enhanced eNOS level and NO production, AHV demonstrated an optimal effect on HUVEC growth and migration, facilitating re‐endothelialization. Furthermore, to initially verify the biocompatibility of each coating, live and dead staining of HUVECs was carried out. The results (Figure S4) showed that the cells grew well on each surface with limited dead cells, demonstrating the proposed coating had no obvious cytotoxicity.

Subsequently, considering the high risk of thrombotic occlusion posed by the fast platelet adhesion on the stent surface, the platelet adhesion suppression on the proposed AHV coating was further evaluated. As shown in the SEM images (Figure 4I), the bare substrate showed no inhibition of adherent platelets. In contrast, the coated groups demonstrated significant suppression of platelet adhesion, with the AHV coating showing only a small number of adherent platelets. This notable platelet inhibitory effect was achieved through the long‐term production of NO and the presence of the anticoagulant heparin. To investigate the related mechanism, the cyclic guanylate monophosphate (cGMP) level in platelets was tested, which is highly associated with antiplatelet activity (Figure 4J). The data showed that the AHV coating elevated the cGMP synthesis (27.53 ± 3.07 nM). Furthermore, a competitive adhesion between HUVECs (green signal) and platelets (red signal) was performed. As depicted in Figure 4K,L and Figure S5, significant platelet adhesion and limited EC adhesion were found in the bare group. After surface modification, there was an increasing trend in EC adhesion. Specifically, although V and AV groups increased the EC adhesion, the platelet amount remained high, potentially due to the cationic nature of the surface. On the other hand, the heparin‐containing groups (HV and AH) displayed a decreased platelet amount, attributed to the shielding of cationic surfaces and the strong anti‐coagulant effect of heparin. Notably, AHV had the highest EC amount with the lowest platelet adhesion. It should be noted that HV exhibited similar EC adherence and platelet repelling trends as AHV, but from the above EC growth experiments, we found that the HV group performed worse than the AHV group in EC proliferation and migration (key elements for rapid re‐endothelialization). Altogether, these findings highlight the favorable combination of enhanced EC adhesion and reduced platelet adhesion on the AHV coating, suggesting its potential as a promising approach for promoting re‐endothelialization and preventing thrombotic complications.

#### 2.3.3 Long‐Term SMC Inhibition

**FIGURE 5:**
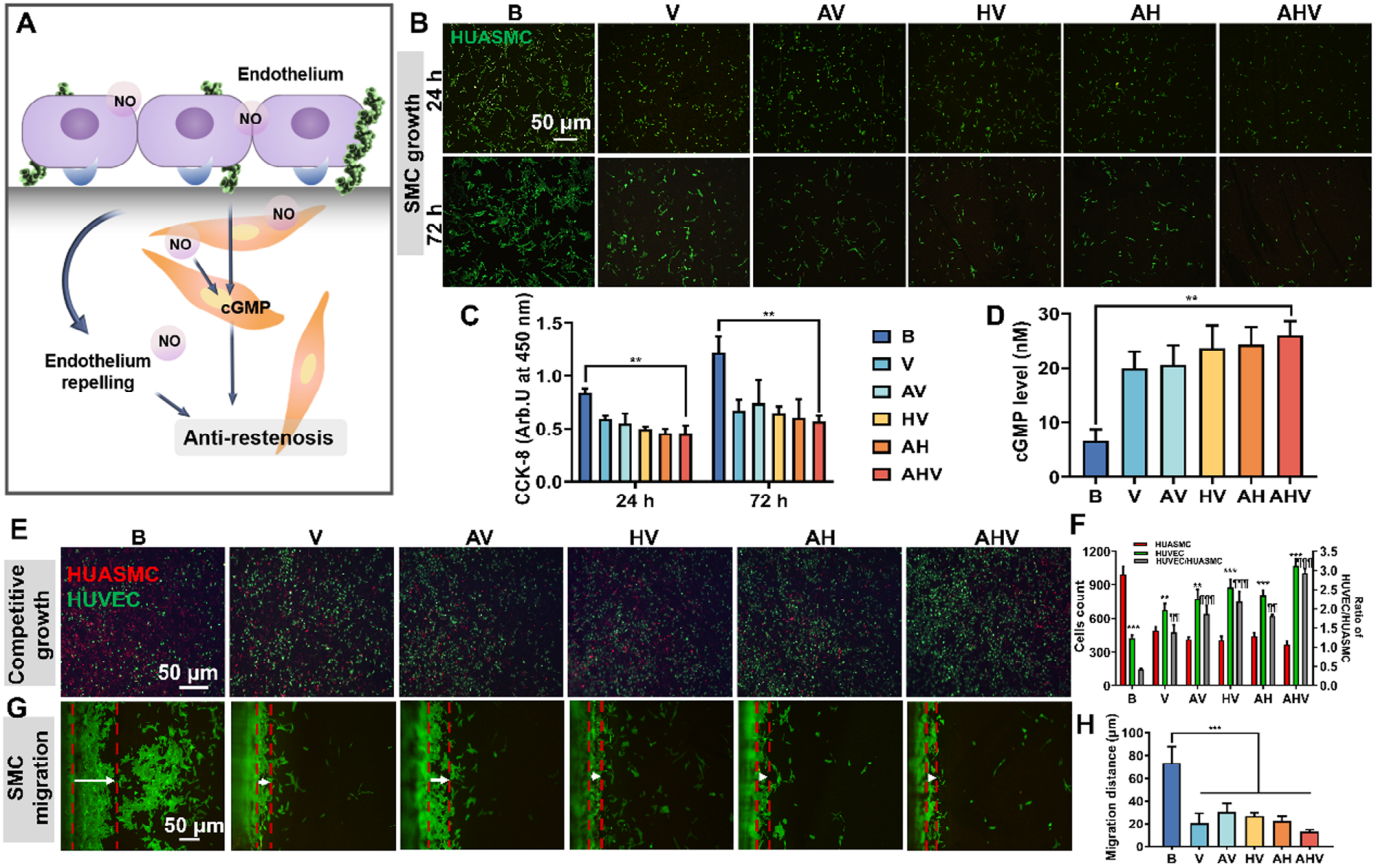
Long‐term SMC inhibition. (A) Illustration of SMC inhibition. (B) Adhesion and (C) proliferation of HUASMCs cultured in the presence of NO donors. Scale bars: 50 µm. (D) cGMP expression after incubation for 72 h with NO donors. (E) Images of competitive adhesion between HUVECs (green) and HUASMCs (red) for 72 h. Scale bars: 50 µm. (F) The ratio of HUVECs/HUASMCs on different coatings. *, **, ***(HUVECs vs HUASMCs) and ¶, ¶¶, ¶¶¶ (vs. 316 L SS) remarked *p* < 0.05, 0.01 and 0.001. The AHV coating has the highest HUVECs/HUASMCs ratio, indicating its potential to support HUVECs and suppress HUASMCs. (G) Fluorescence images of migrated HUASMCs and (H) migration distance of HUASMCs. Scale bars: 50 µm. Data were displayed as mean ± standard deviation (SD) (*n* = 3). *p < 0.05, **p < 0.01, ***p < 0.001. NO: nitric oxide, cGMP: cyclic guanylate monophosphate, HUASMC: human umbilical artery smooth muscle cells, B: bare 316L SS, V: DA/HD‐Cu‐VEGF, AV: DA/HD‐Cu‐arginine‐VEGF, HV: DA/HD‐Cu‐heparin‐VEGF, AH: DA/HD‐Cu‐arginine‐heparin, AHV: DA/HD‐Cu‐arginine‐heparin‐VEGF, HUVEC: human umbilical vein endothelial cell, CCK‐8: cell counting kit‐8.

The over‐growth and excessive migration of SMCs induces extracellular matrix deposition and leads to neointimal hyperplasia and in‐stent restenosis [21, 33]. The mechanism of SMC inhibition is attributed to the endothelial barrier repellence and increased expression of cGMP by NO and heparin (Figure 5A). We further studied the inhibitory effect of the AHV coating on SMC growth. In Figure 5B,C, the bare surface displayed significant green fluorescence of adhered cells, while the coated samples showed limited cell growth. Meanwhile, the AHV coating significantly promoted the cGMP level (26.21 ± 2.14 nM) compared to the bare sample (6.67 ± 1.51 nM) (Figure 5D). To investigate the competitive growth of HUVECs and HUVSMCs, these two cells were co‐cultured on each sample and their growth was traced using green and red fluorescence respectively. After culturing for 24 h (Figure S6), the HUVECs/HUASMCs ratio on B and AHV coating were 0.68 and 2.51, indicating a higher number of HUVECs grown on the AHV coating while the bare surface had more HUASMCs. At 72 h, the AHV‐coated surface was almost fully covered by green signal (referred to as HUVECs) (Figure 5E,F), and it possessed the highest HUVECs/HUASMCs ratio (around 2.94), which was 6.81‐ and 2.11‐fold higher than the B and V groups, and around 1.51‐fold higher than the AV, HV and AH groups. Furthermore, the migration of HUASMCs on different coatings was evaluated. In Figure 5G,H, the bare surface displayed weak inhibition of HUASMC migration (migrated 75.5 µm). However, all the coated groups demonstrated an improved ability to suppress HUASMC migration, and the AHV group showed a significantly reduced distance (12.8 µm). Overall, the AHV coating demonstrated a strong inhibitory effect on SMCs, suggesting its potential to effectively suppress restenosis. These results lead to the conclusion that the biomimetic AHV coating is conducive to ECs and can achieve re‐endothelization to combat SMC‐related complications like hyperplasia and restenosis.

### 2.4 Underlying Mechanism of Improved Vascular Health by the AHV Coating

**FIGURE 6:**
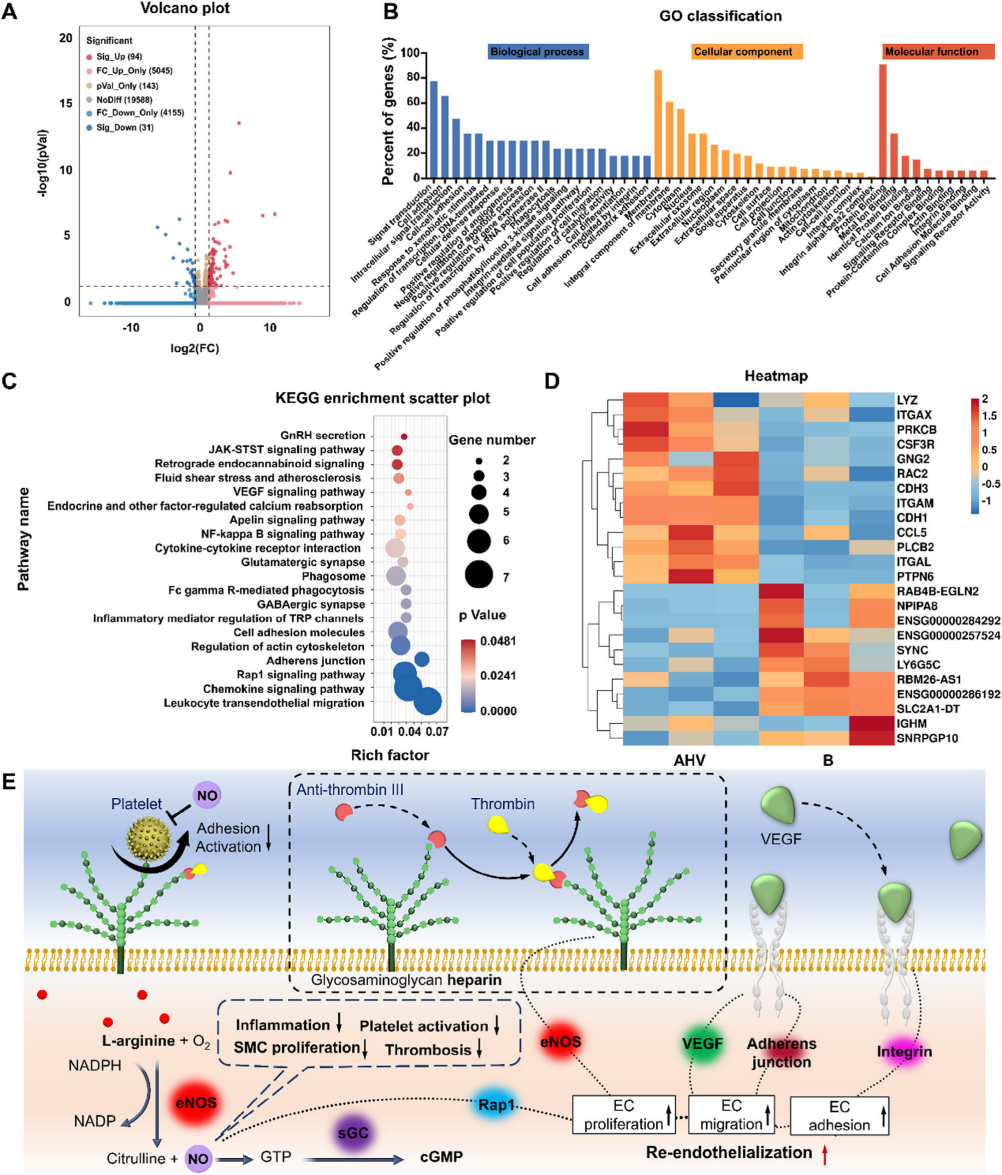
Underlying mechanism investigation by gene sequencing. (A) Volcano graphs. (B) GO classification of DEGs. (C) KEGG enrichment scatter plot. (D) Heatmap evaluation of DEGs (AHV vs. bare). (E) The mechanisms of AHV coating to reestablish a healthy endothelial environment. Data were displayed as mean ± standard deviation (SD) (*n* = 3). AHV: DA/HD‐Cu‐arginine‐heparin‐VEGF, B: bare 316L SS, NO: nitric oxide, O_2_: oxygen gas, NADPH: nicotinamide adenine dinucleotide phosphate hydrogen, NADP: nicotinamide adenine dinucleotide phosphate, eNOS: endothelial nitric oxide synthase, GTP: guanosine triphosphate, sGC: soluble guanylyl cyclase, cGMP: cyclic guanylate monophosphate, Rap1: ras‐proximate‐1, EC: endothelial cell.

To investigate the underlying mechanism of the AHV coating in regulating EC behaviors, a gene sequencing experiment was conducted. Figure 6A illustrates significant gene regulations observed in the AHV‐coated groups compared to the bare samples. The gene ontology (GO) classification analysis of the differentially expressed genes (DEGs) (Figure 6B) revealed that the AHV coating upregulated various biological processes conducive to endothelialization, including cell migration, cell differentiation, angiogenesis, integrin‐mediated adhesion, integrin binding, and cell adhesion binding, etc. Subsequently, a KEGG pathway analysis was performed toward these DEGs (Figure 6C), highlighting pathways positively correlated with the homeostasis of endothelium regulated by the AHV coating. Notably, the Rap1 pathway (known to regulate the endothelial barrier function and NO release), adherens junction pathway (involved in VE‐cadherin‐mediated endothelial integrity) and the VEGF pathway (crucial for vascular development and re‐endothelialization) were activated [34]. Additionally, cell adhesion molecules, and cytokine–cytokine receptor interaction that benefits angiogenesis showed increased activity. The heatmap (Figure 6D) further depicted the changes in DEGs, revealing upregulated genes associated with cell adhesion, including ITGAL, ITGAM, CDH1, CDH3, and PTPN6. Increased expression of ITGAX, which belongs to the integrin family, indicated enhanced VEGF‐mediated EC capture ability [35]. The activation of PLCB2 was conducive to eNOS regulation and NO production [36]. Moreover, the expression of GNG2, CCL5, and RAC2 suggested potential angiogenesis ability conferred by the AHV coating, while the high level of CSF3R exhibited a protective effect for the injured heart [37].

Our findings shed light on the molecular mechanisms behind the AHV coating's positive effects on EC regulation. Such effects endow the AHV coating with preferential regulation over ECs when competing with platelets and SMCs for cell adhesion and growth (Figure 6E). This priority control creates an EC‐friendly environment, where an endothelial layer can wrap around the stent and form a natural protective barrier. The intact endothelium further prevents platelets from adhering to the stent surface, thereby reducing the risk of vascular blockage. Simultaneously, the rapid growth of ECs helps to repair the mechanical damage at the endothelium, greatly inhibiting SMC migration from the injured sites toward the lumen and avoiding luminal stenosis. These findings highlight the potential of the AHV coating to facilitate endothelialization and create a favorable environment for vascularization. By accelerating the blood vessel repair, the AHV coating offers promising prospects for applications in vascular tissue engineering and regenerative medicine.

### 2.5 Ex Vivo Anti‐Thrombosis Investigation of the AHV Coatings

**FIGURE 7:**
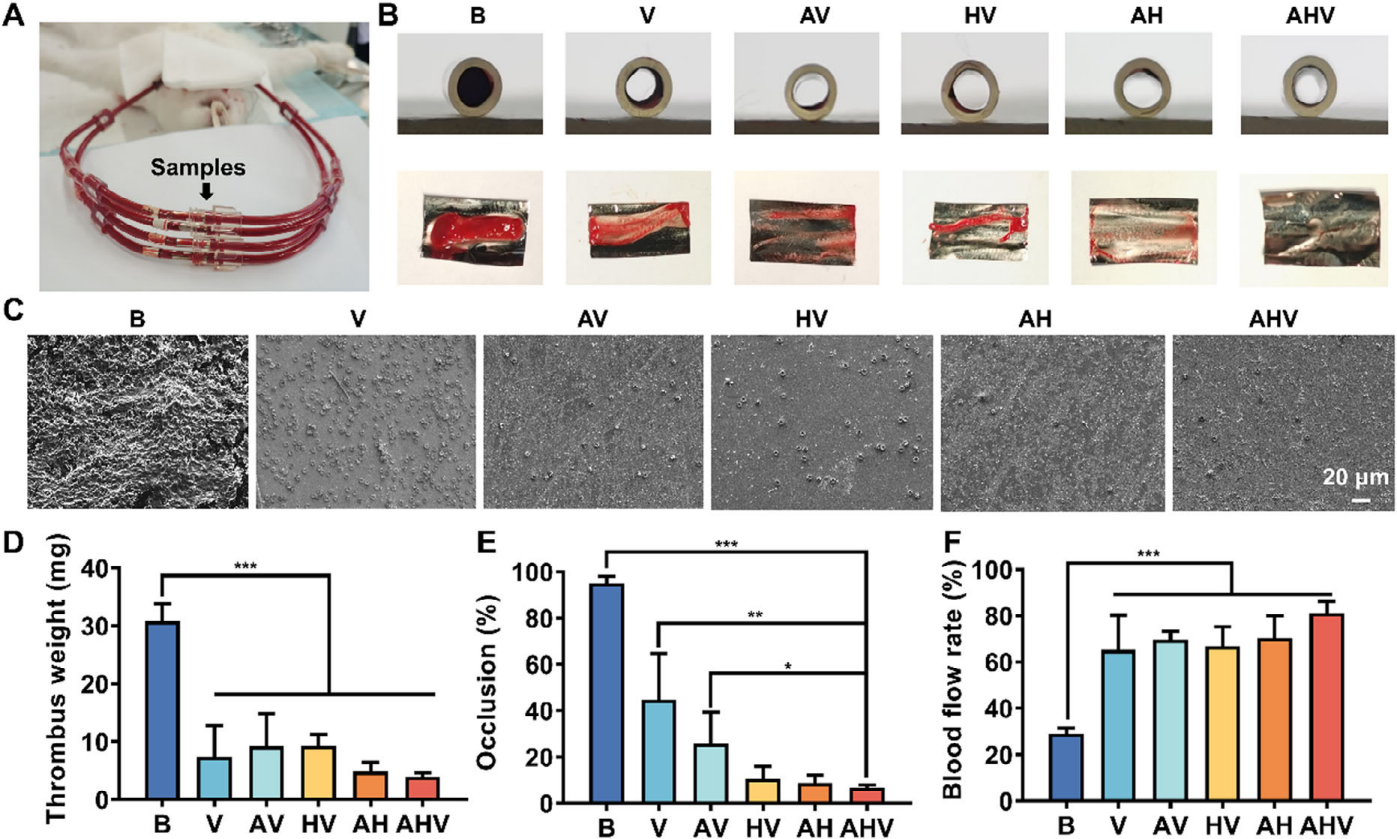
Ex vivo anti‐thrombosis investigation of the AHV coatings. (A) Ex vivo arteriovenous circulation device. (B) Cross‐sections of foil tubes coated with different coatings show the thrombosis formation. (C) Representative SEM images of adherent platelets. Scale bars: 20 µm. (D) Percent occlusion of circuits and (E) weight of thrombus. (F) Blood flow rate. Data were displayed as mean ± standard deviation (SD) (*n* = 4). *p < 0.05, **p < 0.01, ***p< 0.001. B: bare 316L SS, V: DA/HD‐Cu‐VEGF, AV: DA/HD‐Cu‐arginine‐VEGF, HV: DA/HD‐Cu‐heparin‐VEGF, AH: DA/HD‐Cu‐arginine‐heparin, AHV: DA/HD‐Cu‐arginine‐heparin‐VEGF.

To demonstrate the anti‐thrombotic and anti‐platelet functions of the AHV coating, an arteriovenous shunt experiment was conducted (Figure 7A). The photographs of the stented lumen revealed that the bare sample was completely blocked by the blood clots (Figure 7B). On the contrary, the coated samples showed reduced blood clots, with the AHV group showing almost no thrombosis on its wall. After observation by SEM, it could be found that numerous platelets were found on the bare surface, while the AHV coating exhibited minimal platelet adhesion (Figure 7C). Furthermore, the quantitative data (Figure 7D) demonstrated a decreasing trend in the weight of thrombus in each coated group (V: 8.83 mg, AV: 9.23 mg, HV: 9.30 mg, and AH: 4.85 mg), and the AHV‐coated sample had the lowest thrombus weight (3.90 mg) while the bare surface had the highest (30.8 mg). Additionally, Figure 7E demonstrated that the bare sample had an extremely high occlusion rate (95.01%), indicating complete blockage of the circuit, whereas the AHV coating significantly reduced the percentage of occlusion to 6.69%. It is worth noting that the V and AV groups exhibited more thrombus formation and higher occlusion rates among all coated samples. This may be attributed to the increased surface adhesion caused by the positively charged components such as DA/HD and arginine. In the HV, AH, and AHV groups, the incorporation of Hep likely contributed to shielding the surface cations and improving blood compatibility. The reduction in thrombus formation and occlusion rates has a positive impact on the blood flow. The blocked bare samples allowed only 29.16% of the blood flow, while the AHV‐coated samples restored 81.06% of blood flow (Figure 7F). Overall, as evidenced by the near absence of occlusive thrombosis, we can conclude that the AHV coating possessed improved anti‐thrombogenicity.

### 2.6 Long‐Term Anti‐Restenosis Efficacy

**FIGURE 8:**
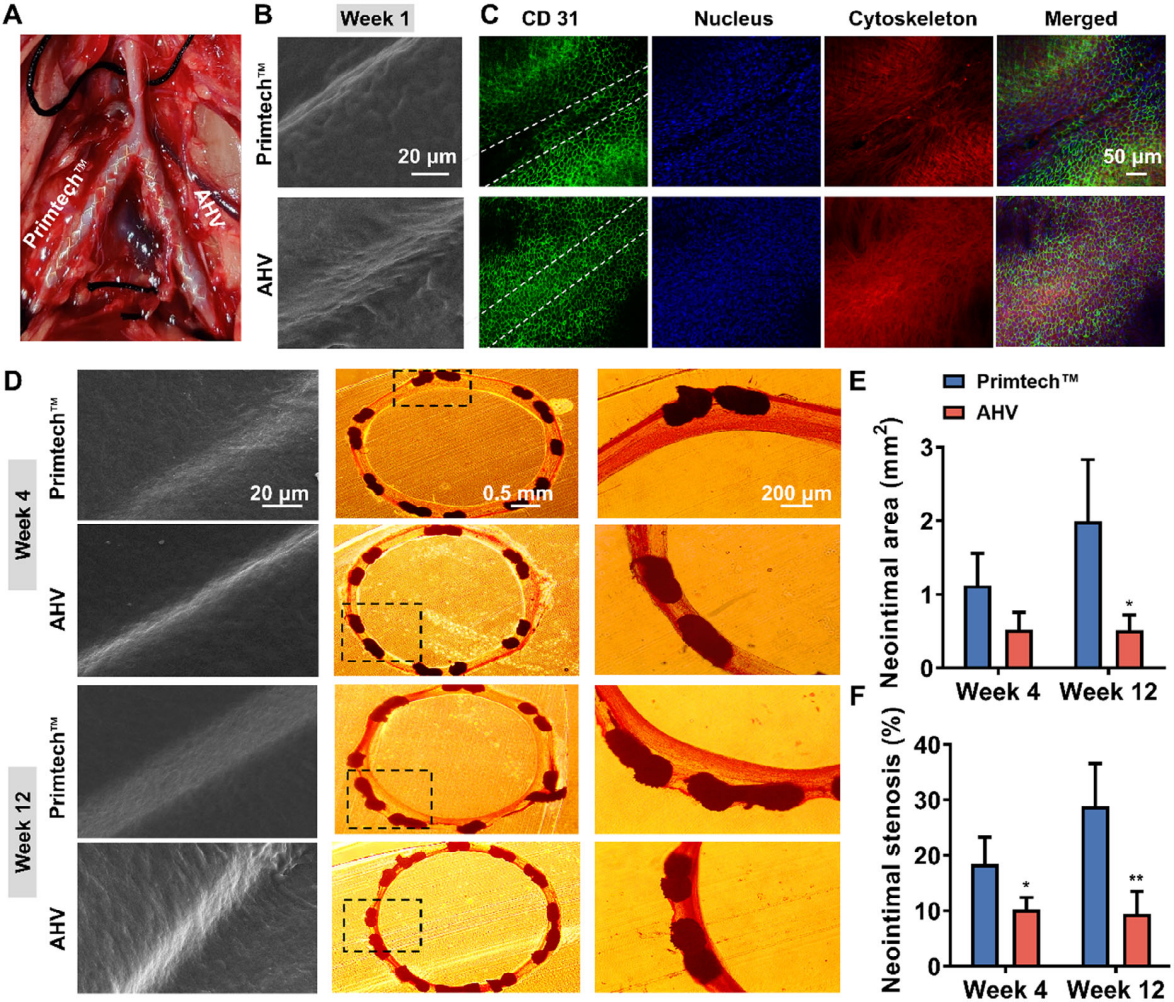
Long‐term in vivo anti‐restenosis effect of AHV coating. (A) Representative image showing stent implantation in a rabbit model. (B) Representative SEM images of implanted stents after implantation for 1 week. Scale bars: 20 µm. (C) CD31 immuno‐fluorescence (green), phalloidin (red) and 4′,6‐diamidino‐2‐phenylindole (DAPI, blue) staining showing re‐endothelialization of 1‐week implanted stents. Scale bars: 50 µm. (D) Representative SEM images and Van Gieson's staining on restenosis of different CVSs. Scale bars: 20 µm, 0.5 mm and 200 µm. (E) Neointimal area and (F) stenosis analysis. Data were displayed as mean ± standard deviation (SD) (*n* = 7). *p < 0.05 and **p < 0.01. AHV: DA/HD‐Cu‐arginine‐heparin‐VEGF, CD31: cluster of differentiation 31.

The results presented above demonstrated that the AHV coating promoted rapid EC adhesion, and inhibited platelet adhesion and SMC growth through the synergistic effect of VEGF, arginine, and heparin. To further investigate the impacts of AHV coating on re‐endothelialization and restenosis prevention, in vivo implantation experiments were carried out (Figure 8A). Following stenting, the rabbits that survived were in a healthy state and could move around and eat as usual. To assess rapid re‐endothelialization, SEM observations and CD31 immunofluorescence staining (a vascular endothelial marker) were performed after 1 week of implantation. In Figure 8B, endothelium‐like cell growth was observed on the AHV‐coated stents, while thromboid erythrocyte deposition was observed on the commercial Primtech stent. Subsequent fluorescence images confirmed endothelium coverage, with red fluorescence indicating cytoskeleton, a green signal representing CD31 expressed by the vascular ECs, and a blue signal indicating cell nuclei. Figure 8C shows that the Primtech stents had limited EC coverage, whereas the AHV‐coated stents demonstrated a continuous green signal, suggesting the AHV coating promoted the regeneration of a new EC layer. At 4 and 12 weeks, SEM images showed that the ECs aligned in the direction of blood flow on the AHV‐coated stents, indicating that the cells on the implanted stents responded to shear stress and adapted to the vascular microenvironment to reconstruct the vascular structure (Figure 8D). Histomorphometric analysis further demonstrated that obvious intimal hyperplasia happened in the Primtech stents, whereas the AHV‐coated stents significantly reduced the neointimal hyperplasia. Specifically, the AHV‐coated stents exhibited a remarkable decrease in the mean neointimal area (Figure 8E) (0.58 mm^2^ in 4 weeks and 0.52 mm^2^ in 12 weeks) compared to the bare stents (1.12 mm^2^ in 4 weeks and 1.99 mm^2^ in 12 weeks). Accordingly, Figure 8F showed the Primtech stents had a high neointimal stenosis ratio at 4 (18.38%) and 12 weeks (28.87%), while a significant attenuation of neointimal stenosis could be found in the AHV stents at 4 (9.45%) and 12 weeks (10.27%). These results demonstrated that the AHV coating established a friendly microenvironment that promotes stent re‐endothelialization, reduces restenosis, and preserves CVS's long‐term patency.

## 3 Conclusion

Surgical interventions involving CVSs are commonly performed procedures for the treatment of CVDs, but issues such as thrombosis and restenosis can limit the therapeutic efficiency of these stents. After stent implantation, a series of cellular events occur in sequence with endothelium damage, and the corresponding treatment for these cellular reactions at different stages will adapt to the physiological responses to the stenting. This project develops a biomimetic stent coating with a double presentation of therapeutic biomacromolecule VEGF and therapeutic gas NO, which spatiotemporally promotes vessel repair. Temporally, the composition of the AHV coating responds to the cellular events after stenting. Specifically, VEGF is able to rapidly capture ECs, while the adherent ECs provide a necessary arena for NOS activation and arginine cleaving. The antioxidant components then mitigate ROS generation and further protect the physiological activity of NO, thereby reversing the inflammatory situation. Spatially, the rapid endothelial wrapping and continuous NO production on the stent can inhibit inappropriate cell behaviors, relieve vascular lumen narrowing and thrombus deposition, restore blood flow, and reshape a healthy endothelial environment.

Our results demonstrated that the AHV coating could reduce ROS levels and facilitate anti‐inflammatory M2‐type macrophage polarization; it could also produce NO under any condition, promote the fast adhesion and growth of ECs and efficiently reduce the platelet aggregation and suppress SMCs. Further animal experiments showed that the AHV‐coated stents demonstrated enhanced re‐endothelialization, anti‐thrombus and anti‐hyperplasia. Consequently, the AHV coating had good potential to be applied in CVSs as it considered the chronological and territorial responses by providing dual delivery of VEGF and NO to target vascular repair mechanism. It eventually improves the therapeutic effectiveness of CVSs and offers valuable insights into spatiotemporal endothelial restoration, inflammation alleviation, thrombosis and hyperplasia inhibition.

The proposed AHV stent coating shows great promise in addressing cellular events occurring after stent implantation, being able to respond to anti‐inflammation, re‐endothelialization, anti‐thrombosis and anti‐restenosis. Moreover, the simple fabrication process is also conducive to clinical translation, and the catechol structure of dopamine enables AHV coating to mimic the mussel adhesive properties for versatile modification onto different material surfaces, such as nickel‐titanium (NiTi) and magnesium (Mg) [38], which opens up possibilities for enhancing the functional performance of these materials in various applications. However, before advancing toward clinical development, it is important to further investigate in vivo responses (such as allergic reactions) to ensure their safety and biocompatibility. Additionally, the development of real‐time, quantitative technology for monitoring NO in vivo is urgently needed to validate the AHV coating's ability to adapt to dynamic physiological conditions and generate appropriate NO fluxes. We hope that these issues can be refined step by step in our subsequent studies to promote the clinical development of stent coatings.

## 4 Experiment Section

### 4.1 Fabrication and Characterization of the AHV Coating

We prepared the AHV coatings on 316L SS substrates by an organic‐free dipping method. Firstly, the substrates were dipped into 1 mg mL^−1^ DA solution with 2.44 mg mL^−1^ HD and 5 µg mL^−1^ CuCl_2_ at 25°C for 24 h to form the DA/HD‐Cu network [25]. Afterward, we immersed the DA‐Cu coated samples into 1 mg mL^−1^ arginine solution for 24 h. Subsequently, heparin (1 mg mL^−1^) was activated with *N*‐hydroxysuccinimide (0.5 mg mL^−1^) and *N*‐(3‐dimethylaminopropyl)‐*N*‐ethylcarbodiimide (1 mg mL^−1^) in 10 mg mL^−1^ 2‐(*N*‐morpholino) ethanesulfonic acid hydrate (pH 5.5) solution, and then the samples were placed into the above solution for 24 h [2a]. Last, the coated samples were dipped in 0.5 µg mL^−1^ VEGF solution for 12 h at 37°C to fabricate the AHV coating samples.

The possible anti‐ferromagnetic coupling of Cu ions in the AHV coating was analyzed using EPR (Bruker, Germany). The possible reaction mechanism between the DA and Cu molecules was investigated using MALDI‐TOF (Bruker, Germany). The elemental composition was analyzed with XPS (Thermo Scientific NEXSA) [39]. Chemical structures were determined using FTIR (Thermo Scientific NEXSA). The mass of arginine, heparin and VEGF covalently immobilized were quantified using QCM‐D (Q‐sense AB, Sweden) equipment. Briefly, after the coated samples were placed inside a test chamber, an arginine/heparin/VEGF solution was sequentially injected into the QCM‐D system, allowing the solution to interact with the surface of each sample. Then, PBS was used to remove weakly bound arginine/heparin/VEGF once the grafting had reached saturation. The Sauerbrey equation Δ*m* = −C*Δ*f*/*n* (Δ*m* is the mass change, *C* is the mass sensitivity constant, Δ*f* is the frequency change, *n* is the overtone number) was used to calculate the adsorption mass, and the amount of residual mass represents the grafting amount [40]. A contact angle meter (OCA‐20, Dataphysics Instruments, Germany) was used to test WCA. A spectroscopic ellipsometer (M‐2000 V, J.A. Woollam, USA) was used to measure the thickness of each coating. The mechanical stability of the AHV coating was performed using a balloon dilation test. SEM (Tescan VEGA3, Czech Republic) images of the AHV stents before and after expansion were taken to analyze the surface morphology change. By inserting dilated AHV stents into polyvinyl chloride (PVC) tubes and connecting them to a peristaltic pump with PBS flow (200 mL min^−1^) for 30 days, the long‐term stability of AHV coating was examined. Surface morphological change was then detected by SEM [16b].

### 4.2 In Vitro Long‐Term NO Production

The AHV‐coated samples were submerged at 37°C in PBS (pH 7.4) with NO donors (10 mM SNAP and 10 mM GSH, changed every 12 h). After 1, 5, 10, 15, and 30 days, the NO production was measured using a total NO assay kit. To quantify the NO amount from arginine decomposition at the cellular level, the samples were submerged in PBS for 30 days. At 0, 7, 14 and 28 days, the samples were taken out, and then HUVECs were cultured at a density of 5 × 10^4^ cells cm^−2^ without the addition of NO donor. Then, the total NO assay kit and 4‐aminomethyl‐2',7'‐difluorescein diacetate (DAF‐FM DA) were used to quantify and visualize NO production.

### 4.3 In Vitro Biological Properties of the AHV Coating

#### 4.3.1 Assessment of the Inflammation Alleviation

Macrophage cells RAW 264.7 were seeded on each sample at a density of 1 × 10^4^ cells cm^−2^ with LPS (1 µg mL^−1^) and then incubated for 3 days with NO donors. For ROS scavenging, the cells on the sample were collected and analyzed by flow cytometry using an active oxygen kit. For immunofluorescence staining, all samples were washed and fixed, CD86 and CD 206 antibodies were used to label M1 and M2 type macrophages respectively, while TNF‐α and IL‐10 antibodies were used to track the pro‐inflammatory and anti‐inflammatory status of macrophage. All cells were observed using a fluorescence microscope (DM2500, Leica, Germany) [41].

#### 4.3.2 Assessment of the Proliferation of HUVECs and HUASMCs

HUVECs or HUASMCs were seeded (5 × 10^4^ cells cm^−2^) onto B, V, AV, HV, AH and AHV samples respectively with the addition of NO donors. After incubation for 24 and 72 h, samples were fixed with 4% paraformaldehyde and then stained using FITC‐phalloidin for fluorescence observation. Cell proliferation on different coatings was assessed via CCK‐8 assay. For HUVECs, after 2 h of incubation, the adherent cells were counted by Image J 1.8.0 software. After incubation for 72 h, the eNOS expression and NO amount were measured by the eNOS ELISA kit and NO assay kit. To verify NO production from arginine and eNOS elevation by heparin, HUVECs (5 × 10^4^ cells cm^−2^, without the addition of NO donors) were seeded onto each sample with or without eNOS inhibitor L‐NMMA (0.5 mM). After 72 h, the eNOS expression and NO amount were measured by eNOS ELISA kit and NO assay kit. To investigate the mechanism of inhibition of HUASMCs, after incubation for 72 h, the cGMP level was measured by ELISA kit [42].

#### 4.3.3 Competitive Growth of HUVECs Over HUASMCs

Before being seeded onto the B, V, AV, HV, AH and AHV coatings, HUASMCs and HUVECs were labelled with red and green respectively, and these two cells were then mixed in equal volume and seeded at the density of 5 × 10^4^ cells cm^−2^. After incubation for 24 and 72 h with NO donors, cell growth was observed, and the number of adhered cells was calculated [43].

#### 4.3.4 Investigation of the Migration of HUVECs and HUASMCs

The foils were folded symmetrically at right angles along the centerline, with V, AV, HV, AH, and AHV coatings deposited on one side while the other side remained bare. To establish a consistent initial state of cell migration, cells with a density of 4 × 10^5^ cells cm^−2^ were cultured on the naked half surface for 6 h, allowing the formation of a cell layer. Subsequently, the foils were inverted, and the coated side was immersed in the medium for 24 h (with NO donors). To observe cell migration from the starting point (the crease) toward the coated side, the cells were stained with FITC‐phalloidin.

#### 4.3.5 Assessment of Adhesion of Platelets

The Hong Kong Polytechnic University's Ethics Committee (18‐19/70‐BME‐R‐GRF) authorized the use of the Sprague Dawley rat blood (male, 3.5–4.5 kg). The blood was centrifuged (1500 rpm, 15 min) to obtain platelet‐rich plasma (PRP). The PRP was diluted to 3 × 10^4^ cells per well, and then the B, V, AV, HV, AH and AHV samples were incubated in PRP for 2 h with NO donors, followed by glutaraldehyde (2.5 wt%) fixation overnight with gradient dehydration and dealcoholization. The adherence of platelets on each sample was observed by SEM. For cGMP expressions, Triton‐X was added into the PRP incubation solution, and the supernatant was analyzed by a cGMP enzyme‐linked immunosorbent assay (ELISA) kit [2, 44].

#### 4.3.6 Competitive Adhesion of HUVECs and Platelets

HUVECs and platelets were labelled with cell tracker green and red respectively and were then mixed in equal volumes (3 × 10^4^ cells cm^−2^) and seeded onto each sample with NO donors. After incubation for 2 h, a fluorescence microscope was used to observe the cell adhesion on the samples, and the number of adhered cells was calculated by Image J 1.8.0 software.

#### 4.3.7 Underlying Re‐Endothelialization Mechanism Elucidation

RNA sequence analysis was carried out to study the possible mechanism of enhanced endothelium repair influenced by the AHV coating. Briefly, HUVECs (5 × 10^4^ cells cm^−2^) were seeded onto the bare and AHV surface with NO donors and incubated for 3 days. Afterward, total RNA was extracted by Trizol reagent and analyzed by the Illumina hiseqx‐10 platform. GO enrichment and KEGG analysis were performed using the cluster Profiler R package [10c].

### 4.4 Ex Vivo Anti‐Thrombogenic Investigation of the AHV Coatings

A rabbit arteriovenous shunt model was established with the authorization of the Ethics Committee of the Tenth Affiliated Hospital, Southern Medical University (K2022‐01‐011‐014). The B, V, AV, HV, AH and AHV foils were curled and fixed in the PVC tubes. Six 3.5‐4.0 kg male New Zealand white rabbits were used in this experiment, with four shunted PVC tubes for each rabbit. The left arteries and right veins of the rabbits were exposed after general anesthesia and barbering. The arteries containing the PVC tubes were then connected with the blood transfusion system. After circulation for 2 h, the PVC tubes were harvested. Cross‐sections of each circuit were photographed; SEM was used to observe platelet adhesion, and the occlusion rate was calculated. Moreover, the thrombus was collected for photographing and weighing [8, 45].

### 4.5 Evaluation of In Vivo Therapeutic Efficacy of the AHV Coatings

The Ethics Committee of the Tenth Affiliated Hospital, Southern Medical University (18‐19/70‐BME‐R‐GRF) authorized the in vivo experiment. Twenty‐four male New Zealand white rabbits (four for 1 week, ten for 1 month and ten for 3 months) weighing between 3.5 and 4.0 kg were used. The rabbits were anaesthetized using 1% pentobarbital sodium, and then the bilateral iliofemoral arteries of the rabbits were separated. Commercially used Primtech (bare 316L SS) stents and the AHV‐coated stents were separately implanted onto the opposite sites of the arteries. After 1 week, 1 and 3 months, the stented samples were harvested. For 1 week, fluorescence microscopy and SEM were used to observe the re‐endothelialization of each sample. For 1 and 3 months, each stented artery were divided into two equal parts: one was fixed with 2.5% glutaraldehyde for SEM detection, and the other was fixed with 4% paraformaldehyde and Van Gieson's staining was used to examine the hyperplasia of stent lumen [46].

### 4.6 Statistical Analysis

Data were presented as mean ± standard deviation. Statistical analysis was carried out by *T*‐test applying GraphPad Prism 8.0 software, *, **, *** represented a statistically significant difference (*p* < 0.05, 0.01, and 0.001, respectively). Unless otherwise stated, each experiment was conducted in triplicate.

## Conflicts of Interest Statement

The authors declare no conflicts of interest.

## Supporting information



Supporting Information

## Data Availability

The data that support the findings of this study are available from the corresponding author upon reasonable request.
