# Supplementary material for: Riding a Vascular Time Train to Spatiotemporally Attenuate Thrombosis and Restenosis by Double Presentation of Therapeutic Gas and Biomacromolecules
Source: Exploration (Beijing). 2025 Feb 4;5(2):70004. doi: 10.1002/EXP.70004 (PMC12087407; doi:10.1002/EXP.70004)
Supplement: Supplementary file 1 — Supporting Information [file EXP2-5-70004-s001.docx]

**Riding a vascular time train to spatiotemporally attenuate thrombosis and restenosis by double presentation of therapeutic gas and biomacromolecules**

Jingdong Rao,^‡a,b,c^ Di Suo,^‡a,c^ Qing Ma,^‡d,e^ Yongyi Mo,^a,c^ Ho-Pan Bei,^a,c^, Li Wang,^f^ Chuyang Y. Tang,^f^ Kai-Hang Yiu,^g^ Shuqi Wang,^h,i^ Zhilu Yang,*^d,e^ Xin Zhao*^a,b,c,j,k^

1. Department of Applied Biology and Chemical Technology, the Hong Kong Polytechnic University, Hung Hom, Kowloon, Hong Kong SAR, China.
2. Department of Biomedical Engineering, the Hong Kong Polytechnic University, Hung Hom, Kowloon, Hong Kong SAR, China.
3. The Hong Kong Polytechnic University Shenzhen Research Institute, Shenzhen, China.
4. Dongguan Key Laboratory of Smart Biomaterials and Regenerative Medicine, The Tenth Affiliated Hospital of Southern Medical University, Dongguan, Guangdong, China.
5. Guangdong Provincial Key Laboratory of Cardiac Function and Microcirculation, Guangzhou, Guangdong, China.
6. Department of Civil Engineering, the University of Hong Kong, Pokfulam Road, Hong Kong Island, Hong Kong SAR, China.
7. Cardiology Division, Department of Medicine, The University of Hong Kong, Queen Mary Hospital, Pokfulam Road, Hong Kong Island, Hong Kong SAR, China.
8. Tianfu Jincheng Laboratory, City of Future Medicine, Chengdu, 641400, China
9. College of Biomedical Engineering, Sichuan University, Chengdu, 610065, China
10. Research Institute for Intelligent Wearable Systems, the Hong Kong Polytechnic University, Hung Hom, Kowloon, Hong Kong SAR, China.
11. Research Institute for Future Food, the Hong Kong Polytechnic University, Hung Hom, Kowloon, Hong Kong SAR, China.

* Corresponding author: Dr. Xin Zhao, E-mail: [xin.zhao@polyu.edu.hk](mailto:xin.zhao@polyu.edu.hk); Prof. Zhilu Yang, E-mail: [zhiluyang1029@smu.edu.cn](mailto:zhiluyang1029@smu.edu.cn)

‡ These authors contributed equally


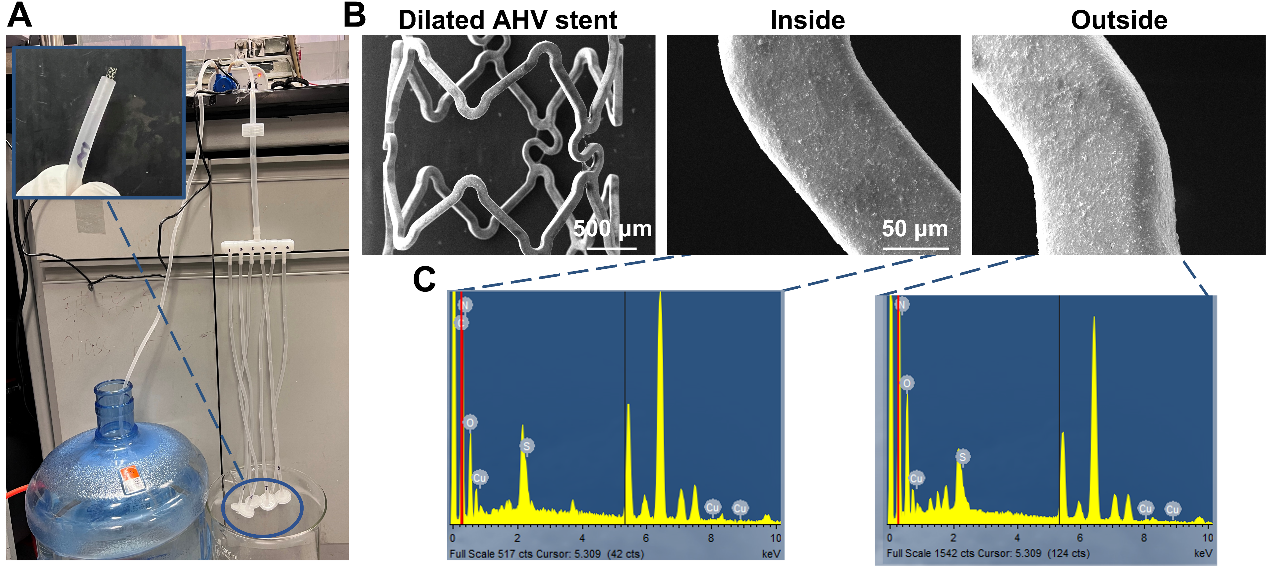


**Figure S1.** **Long-term stability of AHV coating under PBS flow**. (A) Images showing the PBS flush device. (B) Representative SEM micrographs of dilated AHV-coated stents after 30-day of PBS flush. Scale bars: 500 and 50 µm. Note that the coating remained homogeneous after 30-day of flush. (C) EDX spectra of AHV coating. AHV: DA/HD-Cu-arginine-heparin-VEGF, EDX: energy dispersive X-ray.

**
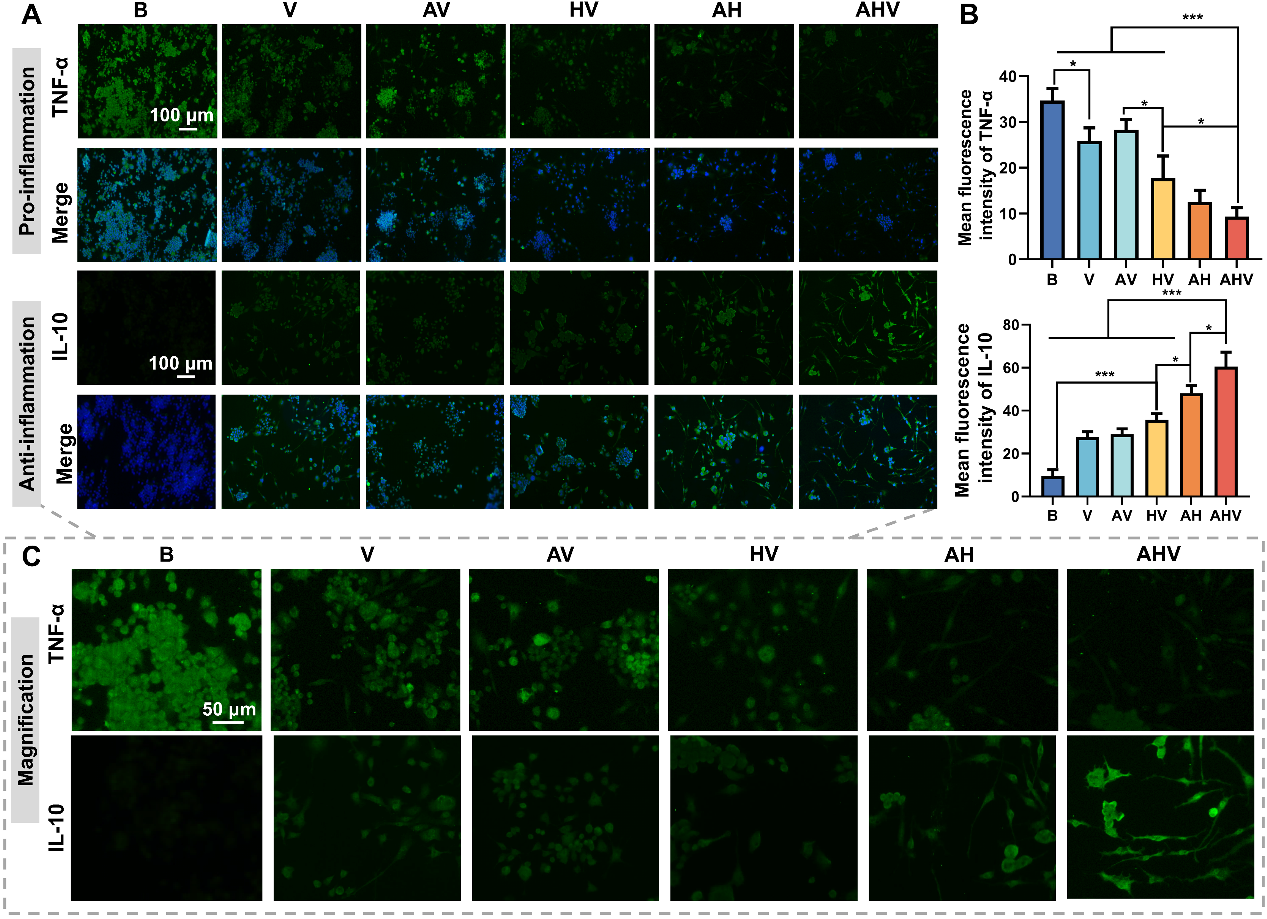
**

**Figure S2. Anti-inflammatory effect of the AHV coating.** (A) Immunofluorescence staining of TNF-α (pro-inflammatory marker) and IL-10 (anti-inflammatory marker) and (B) semi-quantitative data. Scale bars: 100 µm. (C) Magnified image of immunofluorescence of macrophages. M1 type cells are round pancake shaped and M2 type cells are spindle shaped. Scale bars: 50 µm. Data were displayed as mean ± standard deviation (SD) (n = 3). *p < 0.05 and ***p< 0.001. TNF-α: tumor necrosis factor-alpha, IL-10: interleukin 10, B: bare 316L SS, V: dopamine (DA)/hexamethylenediamine (HD)-copper (Cu)-VEGF, AV: DA/HD-Cu-arginine-VEGF, HV: DA/HD-Cu-heparin-VEGF, AH: DA/HD-Cu-arginine-heparin, AHV: DA/HD-Cu-arginine-heparin-VEGF.


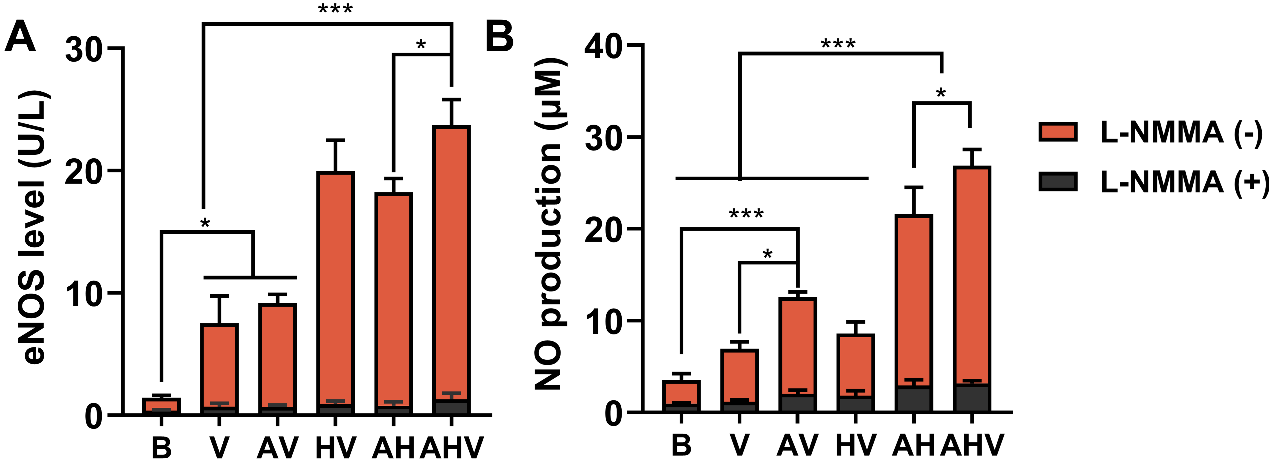


**Figure S3. Validation of NO production effect of arginine.** (A) eNOS inhibitory assay. (B) NO production with or without L-NMMA. Data were displayed as mean ± standard deviation (SD) (n = 3). *p < 0.05 and ***p< 0.001. NO: nitric oxide, eNOS: endothelial nitric oxide synthase, L-NMMA: NG-monomethyl-L-arginine acetate, B: bare 316L SS, V: DA/HD-Cu-VEGF, AV: DA/HD-Cu-arginine-VEGF, HV: DA/HD-Cu-heparin-VEGF, AH: DA/HD-Cu-arginine-heparin, AHV: DA/HD-Cu-arginine-heparin-VEGF.


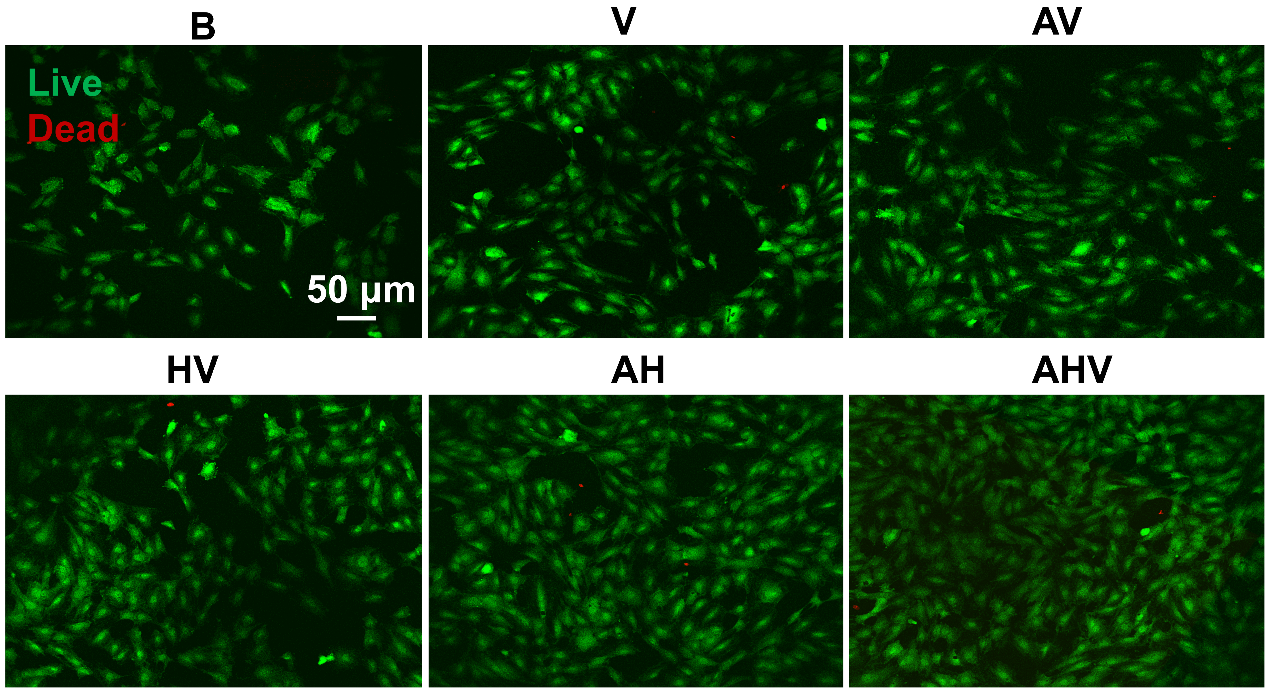


**Figure S4. Live & dead staining of HUVECs (in the presence of NO donors).** Green signal refers to live cells, and red signal refers to dead cells. Scale bars: 50 µm. B: bare 316L SS, V: DA/HD-Cu-VEGF, AV: DA/HD-Cu-arginine-VEGF, HV: DA/HD-Cu-heparin-VEGF, AH: DA/HD-Cu-arginine-heparin, AHV: DA/HD-Cu-arginine-heparin-VEGF.


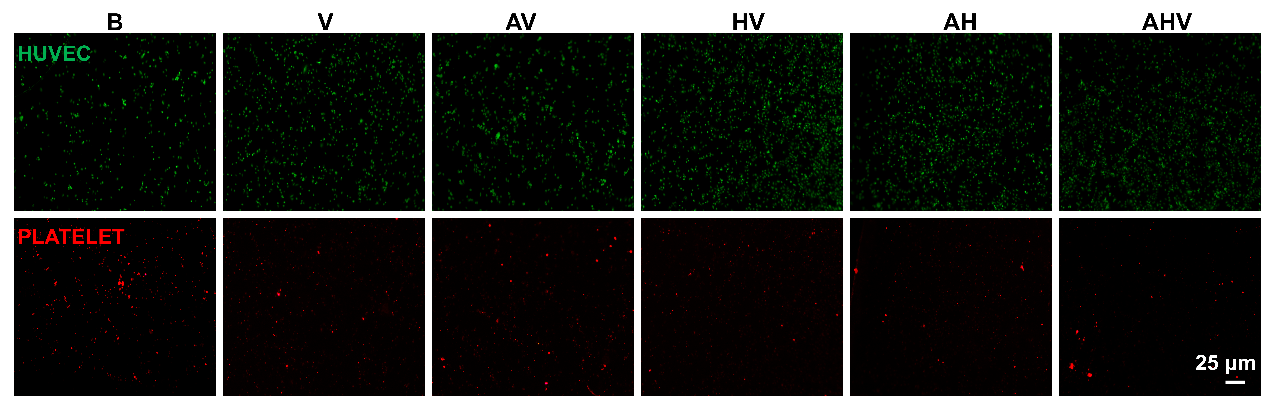


**Figure S5. Fluorescent pictures of co-adhesive HUVECs and platelets at 72 h.** Scale bars: 25 µm. HUVEC: human umbilical vein endothelial cell, B: bare 316L SS, V: DA/HD-Cu-VEGF, AV: DA/HD-Cu-arginine-VEGF, HV: DA/HD-Cu-heparin-VEGF, AH: DA/HD-Cu-arginine-heparin, AHV: DA/HD-Cu-arginine-heparin-VEGF.

**
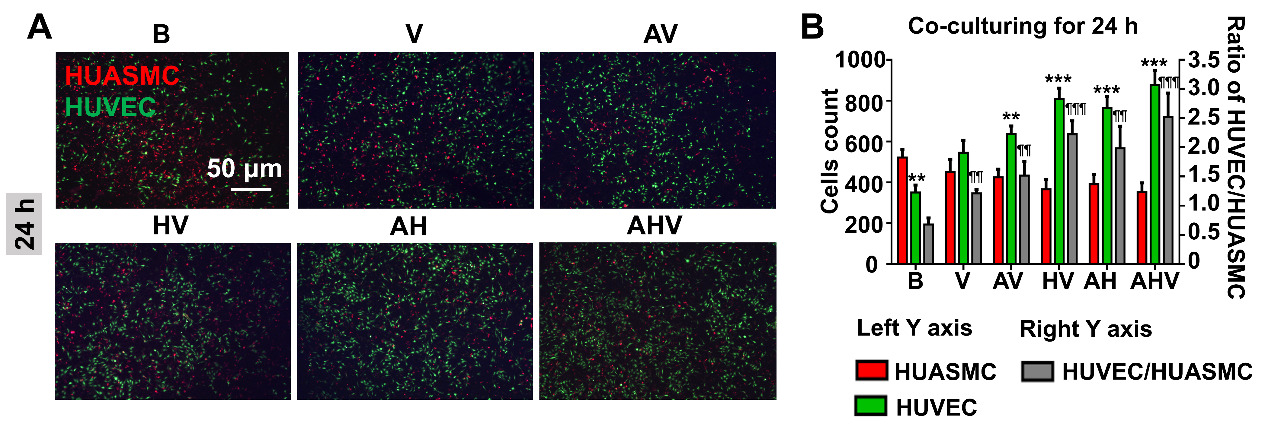
**

**Figure S6.** **Competitive growth of HUVECs over HUASMCs for 24 h.** (A) Images of competitive adhesion between HUVECs (green) and HUASMCs (red). (B) The ratio of HUVECs/HUASMCs on different coatings. Scale bars: 50 µm. *, **, ***(HUVECs vs HUASMCs) and ¶, ¶¶, ¶¶¶ (vs 316 L SS) remarked p < 0.05, 0.01 and 0.001. HUVEC: human umbilical vein endothelial cell, HUASMC: human umbilical artery smooth muscle cells, B: bare 316L SS, V: DA/HD-Cu-VEGF, AV: DA/HD-Cu-arginine-VEGF, HV: DA/HD-Cu-heparin-VEGF, AH: DA/HD-Cu-arginine-heparin, AHV: DA/HD-Cu-arginine-heparin-VEGF.
